# Double IGHV DNA gene rearrangements in CLL: influence of mixed-mutated and -unmutated rearrangements on outcomes in CLL

**DOI:** 10.1038/bcj.2016.49

**Published:** 2016-07-01

**Authors:** B Heyman, A D Volkheimer, J B Weinberg

**Affiliations:** 1 Duke University and Durham Veterans Affairs Medical Centers, Department of Medicine, Durham, NC, USA

B-cell chronic lymphocytic leukemia is a clinically, genetically and molecularly heterogonous malignancy.^1^ During normal B-cell maturation, chromosomal recombination of the immunoglobulin heavy chain V (variable), D (diversity) and J (junctional) gene, and immunoglobulin kappa and lambda light chain V-J gene rearrangement provides the basis for the tremendous immunologic diversity of B cells.^1^ Upon encounter with cognate antigen, B cells can undergo a process of somatic hypermutation during the germinal center reaction in which the recombined VDJ acquire point mutations, thus increasing the affinity for the antigen. Patients with unmutated *IGHV* genes have inferior clinical outcomes when compared with patients with mutated IGHV.^2, 3^ Chromosomal VDJ gene rearrangement is a stochastic, yet highly regulated process. Each B cell undergoes a distinct rearrangement, yielding expression of a unique B cell receptor (BCR). Through the process of allelic exclusion, if the first allelic VDJ rearrangement is productive, VDJ rearrangement does not occur on the second allele. If the first rearrangement is nonproductive, the second allele undergoes VDJ rearrangement to produce a functional BCR. The same principle is followed for light chain rearrangement. The cell undergoes apoptosis if no functional rearrangements are made. Allelic rearrangements can also occur via noncompatible pair of heavy or light chain rearrangement or rescue of deleterious mutations during somatic hypermutation.^4^

Typically, VDJ rearrangement occurs to produce one productive IGHV. However, some chronic lymphocytic leukemia (CLL) cells may lack allelic exclusion and produce two productive rearrangements.^5, 6^ Rassenti and Kipps^5^ reported that doubly productive rearrangements were present in 6 of 108 (5%) samples examined by sequencing complementary DNA derived from patient messenger RNA. Langerak *et al.*^7^ identified 374/2526 (14.8%) of cases with double rearrangements when analyzing DNA and 61/1628 (3.8%) of cases with doubly productive rearrangements when using complementary DNA. Risk, prognosis and outcome where not assessed in studies of Langerak *et al.*^7^

Cerny *et al.*^8^ assessed the DNA of CLL patients and compared a cohort of 17 patients having biallelic IGHV rearrangements with 37 patients having single IGHV rearrangements.^8^ They found that at 3 years, neither OS nor the risk of disease progression was different between the two groups. They did not assess the mutation status of each rearrangement.

We used CLL cell DNA to detect the mutation status of both the productive and nonproductive IGHV rearrangements present in leukemic cells in a prospectively ascertained CLL cohort with treatment and survival outcomes.

Adult patients with CLL from the Duke University and Durham Veterans Affairs (VA) Medical Centers were enrolled into a longitudinal cohort study between 1999 and 2014. CLL diagnosis and Rai staging were according to the NCI Working Group criteria.^9^ Patients were followed clinically for disease progression, need for treatment or death. Primary end points for the study were time to treatment (TTT) and overall survival (OS). OS was from the time of diagnosis to the date of death from any cause and TTT was from the time of diagnosis to the time of first cytotoxic treatment received by the patient. The Duke and VA Institutional Review Boards approved the research, and all enrollees provided written informed consents. Blood CLL cell isolation-enrichment and IGHV mutational analysis were done as we have previously described^10, 11^ using IMGT.^12^ Baseline characteristics were compared using *t*-test, and we used Wilcoxon signed-rank tests and *χ*^2^-test where appropriate. Survival curves were estimated using the Kaplan–Meier method. Statistical differences were tested using proportional hazards, with clinical significance set at *P*<0.05. Patients who did not meet the primary end points were right censored. We used SAS enterprise guide 5.1 (Cary, NC, USA) and JMP Pro 12 (Cary, NC, USA) statistical programs to calculate the statistical outcomes.

Of the 504 CLL patients studied, 441 had one IGHV gene rearrangement (single IGHV; 88%) detected by the polymerase chain reaction amplification of CLL cell DNA. In 62 patients (12%), we noted two IGHV rearrangements (double IGHV). Both rearrangements were detected by RNA analysis in 15 of the 62 (24%) patients in the double IGHV group. This could indicate a lack of allelic exclusion in some of our patients examined here. Age, race, gender, white blood cell at diagnosis, Rai stage, CD38 positivity and cytogenetics were comparable in the single and double groups, however, Zap-70 positivity and IGHV mutation differed between groups (Table 1). Zap-70 positivity was calculated by flow cytometry, using 20% as a cutoff for positivity. Double IGHV patients were more likely to be Zap-70-positive than single IGHV patients (67.3 versus 48.5%, *P*=0.009). Double IGHV patients were also more likely to have at least one mutated heavy chain (74 versus 55%, *P*=0.004).

In our cohort, the single and double IGHV groups were not significantly different in TTT (median 5.4 versus 7.4 years, *P*=NS) or OS (median 16.7 versus 15.8 years, *P*=NS). Patients with single IGHV can be classified as either mutated (m) or unmutated (u). There can be three possible mutation combinations in the double IGHV group: double mutated (mm), mutated–unmutated (mu) and double unmutated (uu). Figures 1a and b display the differences in TTT and OS for the groups. In our cohort, the median TTT for those with mm (not reached), m (11.0 years) or mu (7.9 years) were longer than those with u (3.0 years) or uu (3.2 years). The m, mm and mu groups were statistically different from the u and uu groups. The median OS for those with mm (33.6 years), m (22.2 years) or mu (20.8 years) were longer than those with u (11.9 years) or uu (11.5 years). The m and mm groups were statistically different from the u and uu groups, but the mu group was not statistically different from any other groups, possibly because of the smaller size of the group. We do note that the mu group appears more similar to the m and mm groups than the u and uu groups. Thus, overall, it is solely the presence of mutation that affects survival, not the number of rearrangements (single or double).

In addition to our DNA analyses, we also studied complementary DNA from patient RNA from 12 of the 15 mu patients in our cohort and found only two to be doubly productive. We noted that three patients carried a productive-mutated rearrangement and seven carried a productive-unmutated rearrangement.

According to allelic exclusion, when both *IGHV* genes are rearranged, only one of them should be expressed. Only the expressed rearrangement can be discerned when one analyzes complementary DNA made from patient messenger RNA, but analysis of DNA allowed us to discover both of these rearrangements. For the mm and uu groups, knowing which rearrangement is expressed would not affect disease management as we noted that mm and uu have similar survival to the m and u groups, respectively. When considering the mu group, these patients might express either the mutated or the unmutated rearrangement. Many laboratories analyze leukemia cell messenger RNA when assessing the mutation status. When using messenger RNA, if an unmutated chain were expressed, these patients would be classified as unmutated. However, in using DNA, the mu group as a whole behaved as if it were mutated. By analyzing DNA from these patients, we were able to identify a subset of patients that might otherwise be classified as unmutated when analyzing only RNA, even though their survival is more similar to the mutated group.

Langerak *et al.*^7^ identified 53 mu cases, of which 29 were doubly productive. Of the remaining 24 mu patients, seven carried a productive-mutated rearrangement and 17 carried a productive-unmutated rearrangement. These investigators did not evaluate outcomes. Visco *et al.*^6^ studied only doubly productive rearrangements and found that patients with mu status had worse OS than those with the mm status. We report here that the group of mu patients in our cohort behaved more like m or mm patients than u or uu patients, regardless of which rearrangements were productive.

In our study, 3% of the CLL population had multiple productive IGHV rearrangements. This may be related to a lack of allelic exclusion. Another possibility is that some of these patients may have two distinct CLL cell populations (biclonal CLL). Although CLL has been generally described as a monoclonal disease, there is evidence that biclonality may occur. Kern *et al.*^13^ studied 5523 patients with flow cytometry and identified 76 patients with biclonal CLL. In their analyses, these patients typically had worse outcomes compared with the patients with monoclonal CLL.

In summary, we report the largest single-center study to date that examines double IGHV gene rearrangements and patient prognosis in CLL. Using analysis of CLL cell genomic DNA, we find that the mutational status of the immunoglobulin heavy chain is a very important predictor of TTT and OS (with mutated longer than unmutated). There is no significant difference in survival between patients with single or double IGHV chain rearrangements. However, we document that in patients with double IGHV rearrangements, the presence of at least one mutated *IGHV* gene confers a better prognosis for patients diagnosed with CLL. Therefore, patients who have double IGHV rearrangements with at least one mutated IGHV should be managed as if they have a mutated rearrangement.

## Figures and Tables

**Figure 1:**
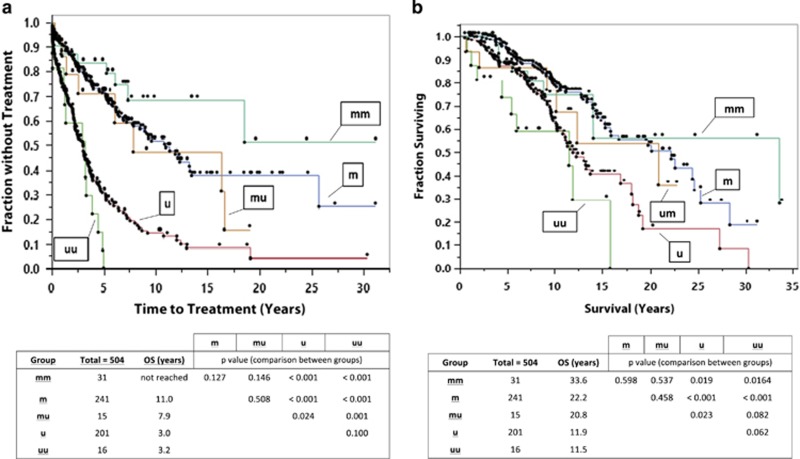
(**a**) TTT for patients with mutated and unmutated IGHV, and statistical analyses among the heavy chain groups. (**b**) OS for patients with mutated and unmutated IGHV, and corresponding statistical analyses among the heavy chain groups. m, mutated IGHV; mm, mutated–mutated IGHV; mu, mutated–unmutated IGHV; u, unmutated IGHV; uu, unmutated–unmutated IGHV.

**Table 1:** Clinical characteristics of patients

*Clinical characteristic*	*Double IGHV*	*Single IGHV*	P*-value*
	*(*n=*62)*	*(*n=*442)*	
Age at Dx, years (median (IQR^a^))	60 (54–69) *n*=61	61 (53–68) *n*=441	NS^b^
			
*Race*	*n*=60	*n*=405	
Caucasian (%)	88.3 *n*=53	85.7 *n*=347	NS
Black (%)	10.0 *n*=6	12.8 *n*=52	NS
			
Male gender (%)	66.1 *n*=41	69.7 *n*=308	NS
WBC at Dx (median (IQR))	20.9 (15.4–29.6) *n*=51	19.5 (15.1–28.9) *n*=359	NS
			
*Rai stage (%)*	*n*=58	*n*=392	
0	69.0 *n*=40	57.4 *n*=225	NS
1	13.8 *n*=8	26.8 *n*=105	NS
2	12.1 *n*=7	7.4 *n*=29	NS
3	0 *n*=0	2.8 *n*=11	NS
4	5.2 *n*=3	5.6 *n*=22	NS
			
ZAP70>20% (%)	67.3 *n*=37/55	48.5 *n*=190/392	0.009
CD38>30% (%)	22.6 *n*=14/62	20.4 *n*=88/432	NS
IGHV mutated (%)	74.2 *n*=46	54.8 *n*=242	0.004
Died (%)	38.7 *n*=24	31.2 *n*=138	NS
			
*FISH*	*n*=45	*n*=313	
13q deleted (only)	42.2 *n*=19	43.1 *n*=135	NS
13q deleted (at all)	55.6 *n*=25	58.5 *n*=183	NS
Normal	24.4 *n*=11	19.5 *n*=61	NS
11q deleted (only)	4.4 *n*=2	3.5 *n*=11	NS
11q deleted (at all)	8.9 *n*=4	11.2 *n*=35	NS
17p deleted (only)	2.2 *n*=1	5.1 *n*=16	NS
17p deleted (at all)	13.3 *n*=6	14.1 *n*=44	NS
Trisomy 12 (only)	8.9 *n*=4	10.9 *n*=34	NS
Trisomy 12 (at all)	15.6 *n*=7	15.3 *n*=48	NS

Abbreviation: WBC, white blood cell.

a IQR signifies ‘interquartile range.'

b NS signifies ‘not significant.'
